# The I. H. A.

**Published:** 1887-07

**Authors:** 


The I. H. A.—We feel sure the members of the I. H. A. must feel very much encouraged in their work by two such successful meetings as its two last. These were eminently practical, useful, and successful; they show the I. H. A. to be a working association. Such work continued from year to year must be productive of the best results for the future of Homœopathy.

We publish in this issue the able address of Dr. Kent, the president, also a brief account of the work of the session. We hope later to publish some of the practical, useful papers presented at the meeting. We have promise of many of them.

